# Biocompatible Cationic Lipoamino Acids as Counterions for Oral Administration of API-Ionic Liquids

**DOI:** 10.1007/s11095-022-03305-y

**Published:** 2022-06-03

**Authors:** Anthony Lai, Nathania Leong, Dan Zheng, Leigh Ford, Tri-Hung Nguyen, Hywel D. Williams, Hassan Benameur, Peter J. Scammells, Christopher J. H. Porter

**Affiliations:** 1 Medicinal Chemistry, Monash Institute of Pharmaceutical Sciences, Monash University, 381 Royal Parade, Parkville, VIC 3052 Australia; 2 Drug Delivery, Disposition and Dynamics, Monash Institute of Pharmaceutical Sciences, Monash University, 381 Royal Parade, Parkville, VIC 3052 Australia; 3 Present Address: Uniquest, General Purpose South Building, Staff House Rd, The University of Queensland, QLD 4072 Brisbane, Australia; 4 Present Address: CSL Limited, 45 Poplar Road, Parkville, VIC 3052 Australia

**Keywords:** absorption, ionic liquids, lipid-based formulations, lipoaminoacids, oral bioavailability

## Abstract

**Purpose:**

The use of ionic liquids (ILs) in drug delivery has focused attention on non-toxic IL counterions. Cationic lipids can be used to form ILs with weakly acidic drugs to enhance drug loading in lipid-based formulations (LBFs). However, cationic lipids are typically toxic. Here we explore the use of lipoaminoacids (LAAs) as cationic IL counterions that degrade or digest *in vivo* to non-toxic components.

**Methods:**

LAAs were synthesised via esterification of amino acids with fatty alcohols to produce potentially digestible cationic LAAs. The LAAs were employed to form ILs with tolfenamic acid (Tol) and the Tol ILs loaded into LBF and examined *in vitro* and *in vivo*.

**Results:**

Cationic LAAs complexed with Tol to generate lipophilic Tol ILs with high drug loading in LBFs. Assessment of the LAA under simulated digestion conditions revealed that they were susceptible to enzymatic degradation under intestinal conditions, forming biocompatible FAs and amino acids. *In vitro* dispersion and digestion studies of Tol ILs revealed that formulations containing digestible Tol ILs were able to maintain drug dispersion and solubilisation whilst the LAA were breaking down under digesting conditions. Finally, *in vivo* oral bioavailability studies demonstrated that oral delivery of a LBF containing a Tol IL comprising a digestible cationic lipid counterion was able to successfully support effective oral delivery of Tol.

**Conclusions:**

Digestible LAA cationic lipids are potential IL counterions for weakly acidic drug molecules and digest in situ to form non-toxic breakdown products.

**Supplementary Information:**

The online version contains supplementary material available at 10.1007/s11095-022-03305-y.

## Introduction

Ionic liquids (ILs) are typically defined as salts with melting points and glass transition temperatures below 100˚C [1–5]. The unique modular structure provided by ILs offers an interchangeable platform of cation–anion pairs [6–9] that has resulted in broad application across a range of ‘task-specific’ ionic liquids (TSILs); i.e. ILs with functionalised properties to perform specific applications [10–13]. In line with the increased general utility of ionic liquids, the use of ionic liquids to enhance drug delivery has also increased in recent years [14–21]. However, despite the wide selection of potential cation–anion pairs, the documented body of work describing IL applications remains focused on a relatively limited pool of IL counterions, especially for cations. For example, a number of studies have focussed on the potential utility of 1,3-dialkylimidazolium cations, such as 1-butyl-3-methylimidazolium (C_4_mim), [22–24] and 1-ethyl-3-methylimidazolium (C_2_mim) [25–29] and these reliably produce ILs with liquid-like properties [30, 31]. Many ILs reported in the literature are composed of organic cations comprising imidazolium or pyridinium analogues [3, 32–35]. Whilst many of these cationic counterions are effective in generating ILs, increased focus on applications in drug delivery has resulted in the need to prioritise biodegradable and non-toxic counterions. Examples of more biocompatible ILs (e.g. biodegradable surfactants and/or biocatalysts) are apparent in allied industries [36–38], but only a few studies have examined the potential for the development of ILs that are biocompatible and biodegradable in the gastrointestinal tract (GIT) and therefore safe for oral drug delivery ([39]). In contrast, many more investigations of ILs in drug delivery have focused on improvements in transdermal drug delivery where issues of acute toxicity are less severe [40–46].

In our previous studies we have focused on the use of ILs as a means to increase the lipophilicity of drug molecules and in doing so to increase drug solubility in traditional lipid based formulations (LBF) [47]. This approach requires the use of lipophilic counterions and in the case of cations paired with weakly acidic drugs, the use of cationic lipids. This however, typically raises questions around the potential toxicity of cationic lipids.

Cationic lipids are commonly utilised as transfection agents in gene delivery studies, often in combination with liposomal vehicles that can be utilised to deliver genetic payloads such as DNA, RNA and other nucleic acids into cells [48–52]. Cationic lipids possess a charged head group, generally provided by tertiary or quaternary amine groups, and a hydrophobic or lipid like tail [53]. They form complexes with negatively charged materials, assisting in eg. association and loading of (anionic) nucleic acids into delivery systems [54–57]. Cationic lipids also promote transfection due to the opposing negative surface charge of cells [58–60]. However, the ability of cationic lipids to interact strongly with most cells types has also led to toxicity concerns that ultimately restrict broader utility. For example, cells do not appear to be able to differentiate exogenous cationic lipids from other positively charged endogenous cell messengers that induce immune responses or cell death signalling pathways, such as endogenous polyamines [61–63]. Cationic lipids may also alter membrane properties and interact with negatively charged components embedded in phospholipid cell bilayers, destabilising the cell membrane [64–66].

Cationic lipids have been employed in the formation of ionic liquids for drug delivery [14, 67] although examples based on longer chain lipid-based cations, including aminoacid esters are more common in the transdermal literature than after oral administration [15, 39]. Nonetheless examples after oral administration are apparent including the use dodecylamine as a counterion for enoxaparin [68] and octadecylamine as a counterion for breviscapine [69], both employed to promote incorporation into oral lipid formulations. The latter examples, however, do not provide for biodegradation. As such, novel cationic lipids that degrade in situ to relatively benign components, and that have the potential to address the issues of toxicity surrounding ILs comprising cationic lipids, are increasingly sought.

**Fig. 1:**
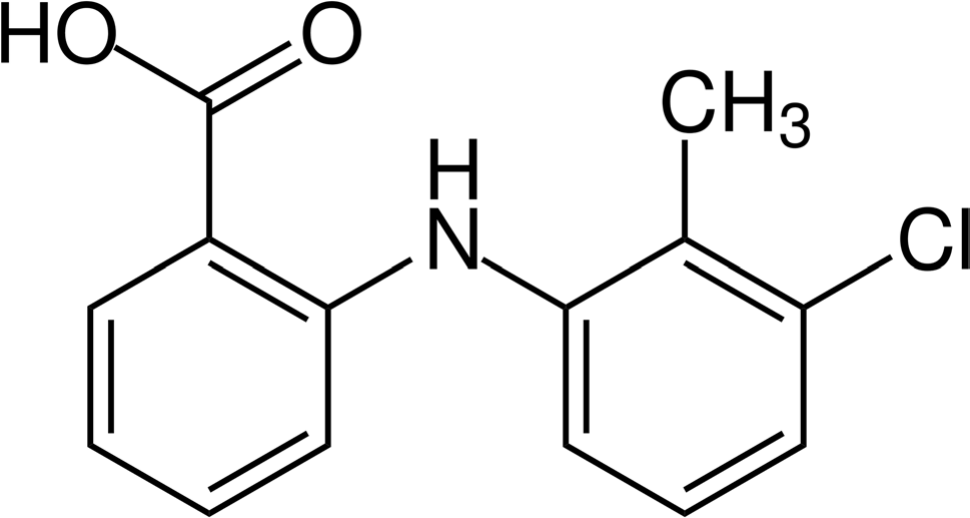
Tolfenamic acid

The current studies have thus explored the potential to form cationic lipid counterions from lipoamino acids (LAA) formed via complexation of fatty alcohols with the carboxylic acid terminal of amino acids. These cationic LAAs have then been complexed with the weakly acidic poorly water-soluble drug (PWSD), tolfenamic acid (Fig. 1) to form tolfenamic acid based, active pharmaceutical ingredient (API) ionic liquids or API-ILs. In general, the cationic LAAs described herein were designed to have some similarity to endogenous lipids in order that the lipophilic ester bond in the LAA may be susceptible to digestion via lipolysis enzymes or more generic esterases in the gastrointestinal tract (GIT). This was expected to result in degradation to fatty alcohols and amino acids, which have considerable safety advantages when compared to typical cationic lipids. Amino acids are ingested through the diet, and it has been reported that aliphatic fatty alcohols (at least between 6 and 22 carbons) are generally safe for oral ingestion [70]. In addition to the API-ILs generated with a digestible LAA counterion we also generated an APL-IL with decylamine (Tol Dec) to provide a non-digestible comparator.

## Materials and Methods

Alanine (Sigma, Castle Hill, NSW, Australia), phenylalanine (Fluka, Pittsburg, PA, USA), hydrogen chloride (HCl) in ether (2.0 M) (Sigma, St. Louis, Missouri, USA), ammonium dihydrogen phosphate (NH_4_H_2_PO_4_) was obtained from Sigma (Castle Hill, NSW, Australia). Formic acid 98 – 100% for HPLC, sodium hydroxide (NaOH) pellets, magnesium sulfate (MgSO_4_); solvents, diethyl ether, methanol (MeOH), chloroform (CHCl_3_) and acetonitrile (ACN) were obtained from Merck (Bayswater, Victoria, Australia). Capmul® MCM and Captex® 355 EP/NF were obtained from ABITEC Corporation (Janesville, Wisconsin, USA); Kolliphor EL was obtained from Sigma (Castle Hill, NSW, Australia).

Trizma® maleate (Castle Hill, NSW, Australia), calcium chloride dihydrate (CaCl_2_·2H_2_O) 99.0% (Castle Hill, NSW, Australia), 4-bromophenylboronic acid, sodium taurodeoxycholate hydrate (NaTDC) (Castle Hill, NSW, Australia) and porcine pancreatin extract (8 × USP specification activity) were purchased from Sigma (St Louis, Missouri, USA). Sodium chloride (NaCl) was purchased from Ajax FineChem (Rosedale, Auckland, New Zealand). Lipoid E PC S (lecithin for egg, ≈ 99% pure phosphatidylcholine (PC)) was obtained from Lipoid GmBH (Ludwigshafen, Germany). Tolfenamic acid (Tol) and decyl alcohol was purchased from Sigma (Castle Hill, NSW, Australia); *p-*toluene sulfonic acid from Fluka (Pittsburg, PA, USA) and toluene from Merck (Bayswater, Victoria, Australia). Organic solvents were used without any pre-treatment. All other chemicals and solvents were of analytical purity or high-performance liquid chromatography (HPLC).

### Synthesis of cationic LAA counterions: decyl alanine ester HCl and decyl phenylalanine ester HCl

**Fig. 2:**
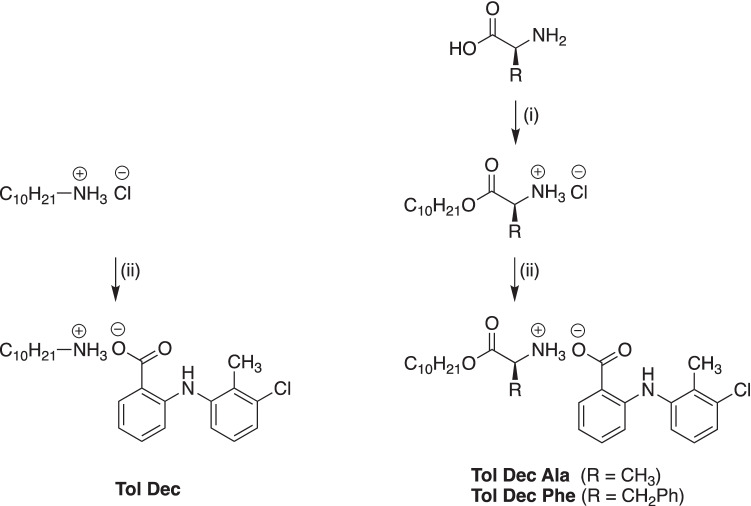
Synthesis of Tol Dec, Tol Dec Ala and Tol Dec Phe; (i) *p*-TSA, toluene, reflux, 24 h then HCl, ether, 0 °C, (ii) Tolfenamic acid sodium salt, MeOH, 0 °C, 1 h

The lipoamino acid (LAA) cations (decyl alanine ester HCl and decyl phenylalanine ester HCl) were synthesized via an esterification reaction. Decyl alcohol (1.0 mmol) and the amino acid (alanine and phenylalanine respectively, 1.0 mmol) were dissolved in toluene (10 mL). *p*-Toluene sulfonic acid (1.3 mol eq) was added to the solution and the reaction was stirred under reflux overnight. Toluene was then removed and the crude product was dissolved in cold diethyl ether. HCl in ether solution was added (2 M, 1.0 mol eq) and the mixture cooled in an ice bath until white crystals formed. The solution was filtered to obtain the HCl salt product of the LAAs (Fig. 2).

### Synthesis of Tolfenamic acid ILs (Tol ILs): Tolfenamate decyl amine (Tol Dec), Tolfenamate decyl alanine ester (Tol Dec Ala) and Tolfenamate decyl phenylalanine ester (Tol Dec Phe)

Tolfenamate decyl amine (Tol Dec), Tolfenamate decyl alanine ester (Tol Dec Ala) and Tolfenamate decyl phenylalanine ester (Tol Dec Phe) were synthesized via a salt metathesis reaction. The reaction is driven by the differential solubility of the salts formed where typically the by-product, sodium chloride (NaCl) precipitates out. Equimolar amounts of the sodium salt of tolfenamic acid and the HCl salt of the cationic LAA were dissolved in methanol (MeOH) and stirred for 1 h. MeOH was then evaporated and the crude product was dissolved in chloroform (CHCl_3_). The organic solution was washed with water to remove the residual by-product, sodium chloride (NaCl). The solution was dried with magnesium sulfate (MgSO_4_) and concentrated to obtain the final product.

## Formulation Preparation

Type IIIA and Type IIIB self-emulsifying drug delivery systems (SEDDS) (as defined by the Lipid Formulation Classification System [71] were freshly prepared as homogenous mixtures of lipid, surfactant and co-solvent. A Type IIIA SEDDS formulation served as a model lipid formulation and is referred to as medium chain formulation—1 (MCF-1). This was composed of 30% Captex® 355, 30% Capmul® MCM, 30% Kolliphor® EL, 10% EtOH (all % values expressed as w/w). An alternate Type IIIB medium chain lipid containing formulations with higher surfactant content was also explored and is referred to as MCF-2. This was composed of 15% Captex® 355, 15% Capmul® MCM, 60% Kolliphor® EL, 10% EtOH (all % values expressed as w/w). A Type IV LBF comprising a homogenous mixture of 50% Kolliphor® EL, 50% EtOH was also employed for the decyl phenylalanine LAA breakdown/digestion studies. This was employed to remove digestible lipids from the formulation thereby magnifying any effects of LAA digestion/breakdown. Tol ILs were dissolved in the MCFs, and concentrations of tolfenamic acid were measured by HPLC.

## Solubility Studies

The solubility of tolfenamic acid and the Tol ILs was determined in the two Type III lipid formulations (described below) via a variation of the shake flask method where Tol ILs were loaded in the lipid formulations incrementally. Tol ILs were loaded into the formulations initially at 10% w/w of the mass of the LBF initially present (e.g., 100 mg of IL added to 1 g of LBF). If the Tol IL completely dissolved, an additional 10% was added to the formulation. The process was repeated until undissolved Tol IL was observed visually. Samples were incubated at 37˚C and left for a number of days until the equilibrium solubility was obtained, defined as < 5% deviation in concentration across consecutive days. Samples were taken at regular intervals and centrifuged (9800 × g, 37˚C, 10 min). The particle-free supernatant was accurately weighed (30 mg) and dissolved in acetonitrile (ACN), followed by further dilution with H_2_O:ACN (20:80, v/v). Samples were then analysed by HPLC. All solubility studies were performed in triplicate. To allow comparison of solubility data between Tol ILs and tolfenamic acid, results are quoted as mg/g of free acid equivalents present in solution in the vehicle.

## *In vitro* lipolysis studies of LBF of Tol ILs

The *in vitro* lipid digestion testing protocol was modelled after the method published by the lipid formulation classification system (LFCS) consortium [72–74]. Digestion buffer (pH 6.5) was made up with 2 mM Trizma® maleate, 1.4 mM calcium chloride dihydrate (CaCl_2_·2H_2_O), and 150 mM sodium chloride (NaCl). To simulate the concentrations of the salts, bile, and phospholipid in the small intestine under fasted conditions a micellar solution of 3 mM sodium taurodeoxycholate (NaTDC) and 0.75 mM Lipoid E PC S was prepared in digestion buffer. Porcine pancreatin extract was prepared for digestion studies at 2400 TBU/mL (1.00 g of pancreatin mixed into 5.0 mL of cold digestion buffer). The activity of pancreatic lipase was expressed in terms of tributyrin units (TBU), where 1 TBU is the amount of enzyme that can liberate 1 mol of titratable FA from tributyrin per min. Porcine pancreatin extract with lower lipase activity was also prepared at 600 TBU/mL (0.25 g mixed into 5.0 mL of cold digestion buffer). The mixtures were centrifuged (2800 × g, 5°C, 15 min) and the supernatant collected to provide the enzyme extract.

Tolfenamic acid and Tol IL were loaded in LBFs at 90% of the equilibrium solubility obtained in the solubility studies. 1.10 g of LBF containing tolfenamic acid or Tol IL was weighed directly into a thermostatically controlled, jacketed glass reaction vessel, and 40 mL of micelle solution was added. The solution was mixed and the pH was buffered and maintained at 6.5 (pH of the small intestine) by the pH stat. Continuous mixing was maintained for 15 min to evaluate the dispersion properties of the LBF. 1 mL samples were taken at 5, 10 and 15 min. Each sample was spun down (9800 × g, 37 °C, 15 min) to separate any precipitate present and the supernatant (aqueous phase). Each phase was collected, followed by a 1 in 200 dilution with H_2_O:ACN, 1:1, v/v. To avoid variability in the quantity of formulation added to each digestion experiment, a control sample was taken after the formulation had been added and dispersed, but without centrifugation, to gain an accurate measure of the total (maximal) concentration of drug in the experiment. The concentration of drug in the aqueous phase digest was subsequently expressed as a % of the measured theoretical target. Digestion was initiated by the addition of 4 mL of pancreatin extract. 0.6 M NaOH solution was utilised in the pH stat to titrate liberated fatty acid and buffer the vessel solution as digestion progressed. Titrants were automatically added via the pH–stat controller and the rate of titrant addition reflected the digestibility of the LBF. 1 mL samples were taken from the dispersion/digestion media at 5, 10, 15, 30 and 60 min after initiation of digestion, and 10 µL of 4-bromophenylboronic acid was immediately added to the samples to arrest lipase activity. Digestion samples received similar treatment to samples taken prior to digestion.

## *In vitro* studies to assess breakdown/digestibility of novel LAA counterions

The *in vitro* lipid digestion testing protocols detailed above were also utilised to assess the breakdown of the decyl phenylalanine ester LAA counterion. An IL formed from the phenyl LAA derivative was employed since the modified LAA possesses a UV active phenyl group and could therefore be assayed quantitatively. For these studies only, Tol Dec Phe was loaded into a Type IV LBF at 37.5% w/w of IL in the formulation i.e. 300 mg of IL in 500 mg of LBF. The required mass of Tol IL was weighed directly into clean screw-top vials, and drug-free lipid formulation was added to target mass loading. Vials were sealed, vortexed and incubated at 37°C for 24 h. The Type IV formulation was prepared as a homogenous mixture of surfactant and co-solvent (50% Kolliphor® EL, 50% EtOH) in the absence of any lipid. This was employed to remove digestible lipids from the formulation thereby magnifying any effects of LAA digestion/breakdown. *In vitro* digestion studies were performed as described above where samples were subsequently analysed for parent LAA (decyl phenylalanine) and the breakdown product phenylalanine.

## HPLC conditions for Tol

Solubility and lipolysis samples were assayed for tolfenamic acid content via HPLC. The mobile phase comprised of 0.1% formic acid in H_2_O:0.1% formic acid in ACN, 20:80 (v/v). Flow rate was 1 mL/min, injection volume 50 μL, and UV detection was at 280 nm. The retention time was 2.7 min, and the concentration range of the calibration standards was 10 – 100 ug/mL. The assay was accurate and precise to within ± 10% of the theoretical concentration across 3 different concentrations.

## HPLC conditions for Tol Dec Phe and Phe

### Decyl phenylalanine (Dec Phe)

Lipolysis samples of Tol Dec Phe were assayed for Dec Phe content via HPLC. The mobile phase comprised of 0.1% formic acid in H_2_O (Mobile Phase A) and 0.1% formic acid in ACN (Mobile Phase B) and was eluted using linear gradient elution. The initial conditions were 80:20, (A/B) v/v decreasing to 40:60, v/v, between 0.00 – 1.00 min, after which the gradient remained at 40:60, v/v, between 1.00 – 3.50 min, followed by a linear gradient to 20:80, v/v, between 3.50 – 4.50 min. The mobile phase was held at 20:80, v/v, between 4.50 – 5.50 min and then immediately returned to 80:20, v/v, which then remained at that volume ratio between 5.50 – 7.50 min. The flow rate was 1 mL/min, injection volume was 50 μL, and UV absorbance was set at 214 nm. The retention time of Dec Phe was 2.5 min, and the concentration range of the calibration standards was 20 – 200 ug/mL. Standard curves were prepared by plotting peak height ratios against known concentration of standards. Calibration stock solutions of Dec Phe were prepared at a concentration of 2000 µg/mL by dissolving Dec Phe in ACN. Standard samples were prepared with H_2_O:ACN, 80:20, v/v. Unknown sample concentrations were calculated from the standard equation y = mx + c, as determined by linear regression of the standard curve. Assay performance was validated using standard measures of linearity, precision, and reproducibility.

### Phenylalanine (Phe)

Mobile phase comprised of 0.1% formic acid in H_2_O (mobile phase A) and 0.1% formic acid in acetonitrile (ACN) (mobile phase B) and was eluted on a linear gradient elution. Mobile phase started at 98% v/v A between 0.0 – 4.0 min. The gradient was reduced to 20% A between 4.0 – 4.5 min, and 20% A was maintained between 4.5 – 5.0 min. The gradient was returned to 98% A between 5.0 – 6.0 min, and 98% A was maintained between 6.0 – 6.5 min. The flow rate was 1 mL/min, injection volume was 50 μL, and UV absorbance was measured at 214 nm. The retention time of Phe was 2.0 min, and the concentration range of the calibration standards was 5 – 200 µg/mL. Standard curves were prepared by plotting peak heights against known concentration of standards. Calibration stock solutions of Phe were prepared at a concentration of 2000 µg/mL by dissolving Phe in H_2_O. Standard samples were then prepared by dilution with H_2_O:ACN, 98:2 (v/v).

Unknown sample concentrations were calculated from the standard equation y = mx + c, as determined by linear regression of the standard curve. Assay performance was validated using standard measures of linearity, precision, and reproducibility.

## Pharmacokinetic studies

All animal experiments were approved by the Monash Institute of Pharmaceutical Sciences Animal Ethics Committee and were conducted in accordance with the Australian and New Zealand Guidelines for the Care and Use of Animals in Research. Male Sprague Dawley rats (260–340 g) were obtained from the Monash Animal Research Platform (VIC, Australia).

One day prior to dosing, the left internal carotid artery was cannulated to facilitate blood collection as described previously [75]. Surgeries were conducted under gaseous anaesthesia using 3% isoflurane in medical carbonox (95% oxygen, 5% carbon dioxide). The cannula (a proprietary BASi Culex cannula, BASi West Lafayette, IN) was externalised by subcutaneous tunnelling to emerge at the back of the neck. Rats were placed in Raturn® metabolic cages connected to a Culex® automated blood sampler (BASi, West Lafayette, IN) to recover overnight and for the duration of the pharmacokinetic study. Animals were fasted overnight and food was returned 6 h post dose. Water was available ad libitum.

On the day of dosing animals were briefly anaesthetised with isofluorane prior to dosing via oral gavage. Animals were dosed with either i) a suspension prepared by dispersing 5.6 mg tolfenamic acid in 0.5% CMC (w/v) and 0.4% Tween 80 (w/v) in 0.5 mL of 0.9% NaCl, ii) a lipid suspension of tolfenamic acid in MCF-2 where 5.6 mg tolfenamic acid was suspended in 125 mg of MCF-2 and dispersed in 0.5 mL water immediately prior to gavage (~ 45 mg/g tolfenamic acid), or iii) 26 mg Tol Dec Ala dissolved in 125 mg of MCF-2 and dispersed in 0.5 mL water immediately prior to gavage (~ 207 mg/g IL or 110 mg/g tolfenamic acid equivalents).

Plasma samples were collected up to 15 h post-dose. At each sampling time, 220 µL of arterial blood was collected directly into heparinised borosilicate vials stored in the chilled Culex® fraction collector. Samples were centrifuged at 2000 x g for 3 min at 4°C, and 50 µL aliquots of plasma were transferred to microcentrifuge tubes and frozen until sample analysis. T_max_, C_max_ and AUC_(0-15 h)_ data were taken directly from the plasma level time curves and AUC calculated via the linear trapezoidal rule. Dose normalised AUC data were calculated using a nominal dose of 5.6 mg/rat to allow comparison of exposure at the same dose, recognising that the IL containing formulation could be administered at a higher dose due to higher solubility.

## Plasma assay for tolfenamic acid

A 5 µL aliquot of internal standard (diclofenac) solution was spiked into each 50 µL plasma sample, and the samples were vortexed for 0.5 min. Acetonitrile (100 µL) was subsequently added to precipitate proteins and samples vortexed and centrifuged at 10,620 x g for 10 min at 25°C. The supernatant from each sample was collected and transferred to HPLC autosampler vials for analysis. Plasma standards were prepared by spiking aliquots (45 µL) of blank rat plasma with 5 µL of tolfenamic acid standard solution and 5 µL of internal standard solution. Plasma standards were subsequently prepared in the same manner as the plasma samples before analysis by LC–MS/MS. Mass spectrometry was performed using a Shimadzu LC–MS/MS 8050 triple quadrupole mass spectrometer (Shimadzu, Japan) coupled to an LC20AD solvent pump system and SIL 20AC HT autosampler (Shimadzu, Japan). The optimized mass spectrometry settings were: detector voltage: 1.1 kV; interface temperature: 350° C; DL temperature: 300° C; heat block: 450°C; nebulising gas flow: 1.5 L/min; drying gas flow: 6 L/min. Analytical separation was performed using a Phenomenex Kinetex 2.6 µm C8 100 Å, 50 × 2.1 mm column at a mobile phase flow rate of 0.3 mL/min. Mobile phase comprised 0.1% formic acid in both solvent A (MilliQ) and solvent B (methanol). Samples were injected onto the column and eluted using a binary gradient: 50%-97% solvent B from 0–1.65 min; 97% B from 1.65–2 min 97–50% B from 2–2.5 min and 50% B from 2.5–3 min. The retention times of tolfenamic acid and diclofenac were 2.23 and 1.99 min respectively.

## Results and discussion

### Solubility studies of Tol ILs in MCF

Previous studies have shown that converting poorly water soluble drugs (PWSD) into lipophilic drug-ILs can increase the drug loading capacity of lipid based formulations (LBFs) [76–78]. For PWSDs that are weak electrolytes, strong acids or bases are commonly used to provide the pairing counterions in pharmaceutical salts in order to promote complete proton transfer. As a rule of thumb complete proton transfer is expected where the ∆pK_a_ between the acid and base counterion pairs is > 2 – 3. [79–82]. The respective pK_a_s of Tol (pK_a_ = 3.66) [83, 84] and the lipophilic amines employed here (pKa ~ 10) [85, 86] leads to a ∆ pK_a_ ~ 6.4, which was expected to promote efficient proton transfer between the drug and amine. As a result, Tol ILs were expected to display more typical behaviour than for example the cinnarizine ionic liquids with fatty acid counterions we have described previously [47]. Consistent with this suggestion Tol readily formed IL with the counterions employed here and no evidence of instability or disassociation was apparent over the period of study. As synthesised the ILs produced had different physical properties. Tol Dec was isolated as a white solid, with a clearly defined melting point of 147 °C (and so sat outside the typical definition of an IL). In contrast Tol Dec Ala (and Tol Dec Phe) were isolated as yellow, viscous semi solid amorphous materials that revealed glass transition temperatures by DSC of -16 and -6 °C respectively (supplementary information).

To determine the equilibrium solubility of the Tol ILs in medium chain LBFs, Tol ILs were first loaded incrementally into MCF-1, starting at 10% mass of IL/mass of LBF and the quantity of IL added increased when complete solution was apparent. As shown in Table I, converting tolfenamic acid to Tol ILs utilising lipophilic amines generally increased drug loading capacity. Tolfenamic acid decyl amine (Tol Dec) and tolfenamic acid decyl alanine ester (Tol Dec Ala) increased the solubility of tolfenamic acid in lipid solution by 2 and 2.5 fold respectively. The greater increase in solubility provided by the Dec Ala counterion when compared to the Dec counterion may reflect the methyl side chain group on the alanine introducing steric bulk, thereby disrupting lattice formation and increasing drug loading capacity. This is consistent with the lower Tg of Tol Dec Ala when compared to the melting point of Tol Dec. Interestingly, the Tol IL with the largest side chain group, Tol Dec Phe (that was employed as a probe in the breakdown studies to allow quantification of Phe) displayed a lower solubility than the free acid, in spite of a lower Tg.

**Table I:**
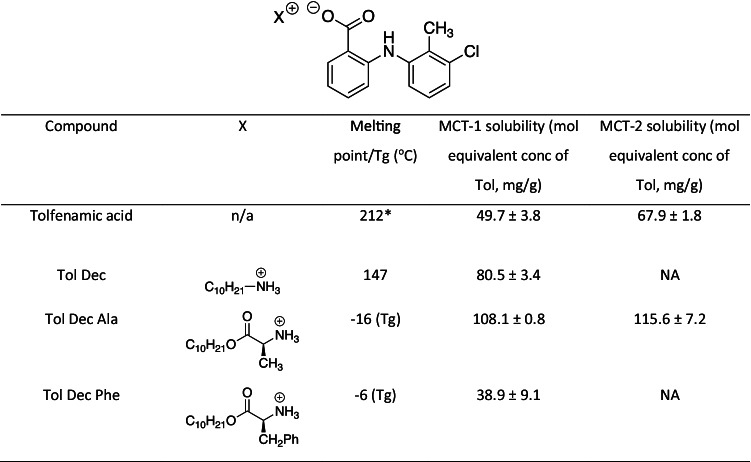
Solubility of tolfenamic acid and Tol ILs in LBF

* M.P of Tol from [87]

### The digestibility of LAA cations under simulated digestive conditions

To evaluate the digestibility of the LAA counterions, the decyl phenylalanine ester LAA was generated in order that the phenylalanine breakdown product could be readily quantified (unlike alanine that does not have a UV chromophore). To facilitate these experiments, tolfenamic acid decyl phenylalanine ester (Tol Dec Phe) was loaded into a relatively poorly digestible Type IV LBF which contains no traditional lipid to ensure that observations of the digestibility of the LAA were not complicated by parallel effects on formulation digestion. The IL loaded formulation was dispersed into simulated intestinal fluid and digestion was initiated by the addition of porcine pancreatin extract (2400 TBU/mL). Samples were routinely taken during digestion and processed to determine the concentration of Dec Phe, and the amino acid breakdown product phenylalanine (Phe).

**Fig. 3:**
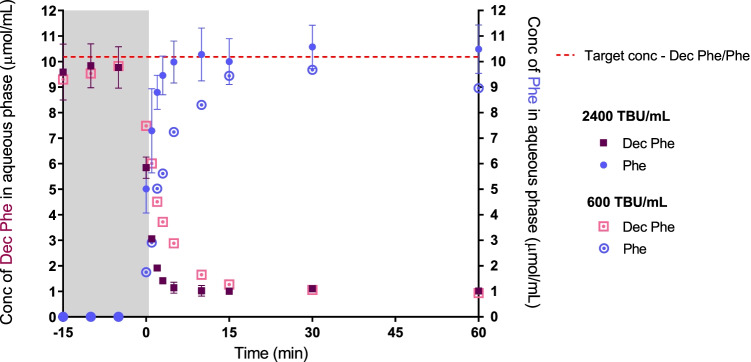
Lipolysis of Tol Dec Phe in a Type IV formulation demonstrating the digestive breakdown of the Dec Phe counterion. The study was split into two phases, an initial dispersion phase, grey area (-15 – 0 min), and a period after initiation of digestion (0 – 60 min). Digestive breakdown of Dec Phe is illustrated by the decreasing concentration of Dec Phe upon addition of porcine pancreatin extract (> 0 min), and a proportional increase in concentrations of phenyalalnine (Phe). The dashed red line shows the theoretical maximal concentration of Dec Phe and Phe (i.e. with no digestion or complete digestion respectively)

As shown in Fig. 3. the concentrations of Dec Phe and Phe measured throughout digestion showed the expected inversely proportional relationship, i.e. breakdown of Dec Phe producing Phe. Figure 3. also reveals a very rapid rate of LAA breakdown (< 5 min) under standard simulated digestive conditions. To better illustrate the breakdown of the Dec Phe, the volume of porcine pancreatin extract added to the medium was reduced four-fold (to 600 TBU/mL, 25% w/v). As expected this reduced the rate of LAA breakdown (digestion of Dec Phe completed at 15 min). These data demonstrate the susceptibility of Dec Phe to enzymatic breakdown under digestive conditions. Since the bulky phenyl group of the Dec Phe counterion did not significantly limit enzyme approach it seems likely that Tol ILs with LAAs such as decyl alanine ester (i.e. which lack the benzyl group) as well as similar LAAs with smaller side chain groups are likely to be susceptible to enzymatic degradation. Notably the products of digestion, amino acids and fatty alcohols are classified as food additives and are expected to present no toxicity risk [88, 89].

## Lipolysis studies of Tol ILs in MCF-1 (Type IIIA formulation)

Having shown that the Dec Phe LAA counterion was readily digestible, subsequent studies sought to evaluate whether LBF containing tolfenamic acid and Tol ILs based on the Dec, Dec Ala and Dec Phe LAA were able to maintain drug solubilisation during dispersion and digestion of the LBF.

**Fig. 4:**
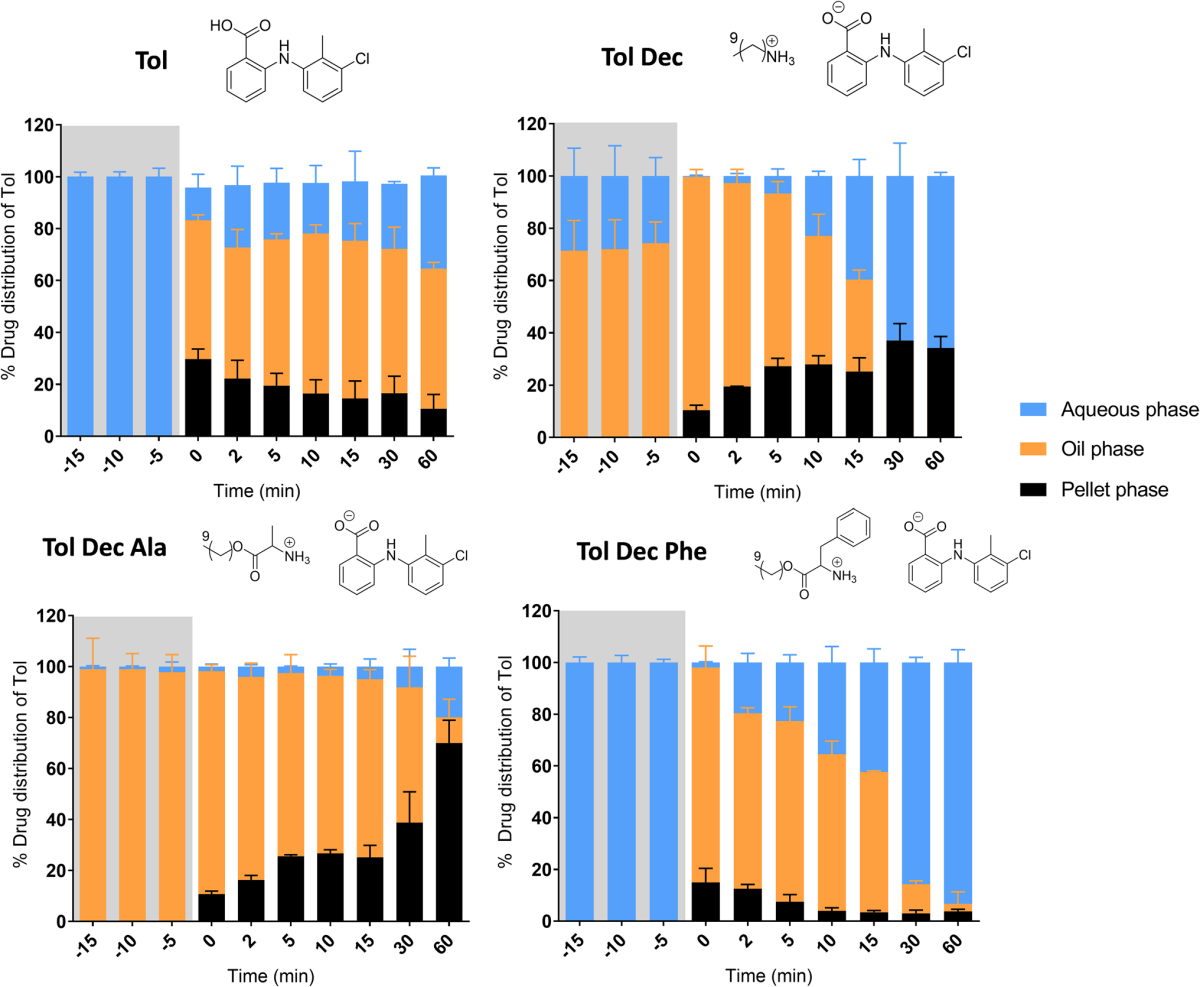
Drug distribution of tolfenamic acid and Tol ILs in MCF during lipolysis studies (Type IIIA) (37 °C, mean ± SD, n = 3). % drug distribution of tolfenamic acid was determined via the concentration of tolfenamic acid in respective phases during dispersion (-15 – 0 min; grey section), and digestion (0 – 60 min)

On initial dispersion in the *in vitro* lipid digestion test, LBF containing tolfenamic acid and Tol Dec Phe resulted in good dispersion and excellent solubilisation of Tol in the aqueous phase (Fig. 4). On initiation of digestion, however, LBF containing tolfenamic acid and Tol Dec Phe generated some precipitated material, a dense oil phase (that settled above the pellet after centrifugation) and limited quantities of Tol in the aqueous phase. Interestingly, as digestion progressed the quantity of Tol in the aqueous phase increased and amounts in the dense oil phase and pellet reduced. This was particularly evident for Tol Dec Phe, where significant transfer to the aqueous phase was seen over time resulting in more than 90% of the drug being recovered in the aqueous phase after 60 min digestion. Similar patterns of distribution were seen for tolfenamic acid however, the changes were not as large.

In contrast, to tolfenamic acid and Tol Dec Phe, dispersion of LBF containing Tol Dec and Tol Dec Ala resulted in the immediate formation of a dense oil phase that contained most of the drug, limiting transfer to the aqueous phase. On digestion, Tol Dec loaded LBF showed behaviour very similar to that of tolfenamic acid and Tol Dec Phe, i.e. a high % of tolfenamic acid present in the dense oil phase at the onset of digestion, but subsequent transfer to the aqueous phase over time. However, unlike tolfenamic acid and Tol Dec Phe, Tol Dec resulted in increased precipitation of tolfenamic acid as digestion progressed. Similar to Tol Dec, Tol Dec Ala also formed a dense oil phase and aqueous phase during dispersion, and separated out into three phases upon digestion. Interestingly, unlike the other Tol IL, in the case of Tol Dec Ala a small quantity of oil phase remained until the end of digestion. More importantly, as the oil phase reduced, instead of drug transferring to the aqueous phase (as it did for Tol Dec and Tol Dec Phe), drug precipitated and was recovered in the pellet. The differences in phase behaviour and solubilisation over time may be related to the digestion of the counterion over time, however detailed analysis of the species present was not undertaken in these experiments.

The differing drug distribution profiles observed for the Tol ILs studied here may be due to the nature of the different Tol ILs loaded into the formulation and in particular the differences in the mass of IL loaded (due to differing solubilities). Thus, the phase behaviour of the more MCF soluble Tol ILs (such as Tol Dec Ala where > 10% w/w of mass of IL/mass of LBF was loaded), may result from the very large quantities of the (dense) IL that could be loaded into the formulation. This in turn may have driven phase separation of a drug rich oil phase during processing. Whilst a proportion of this ultimately transfers to the aqueous phase, probably due to digestion of the counterion, the large quantity of IL loaded dictates that this is incomplete. In contrast, in those ILs that are loaded at much lower levels due to lower solubility (such as Tol Dec Phe) similar processes occur, but the much lower mass of IL that must be processed dictates that transfer to the aqueous phase is more efficient. For the IL with intermediate solubility (Tol Dec) intermediate behaviour is apparent. In contrast to the IL loaded systems, tolfenamic acid behaves more typically and disperses well initially but then precipitates as digestion continues. In this case kinetic changes to the counterion due to counterion digestion do not complicate the solubilisation profile.

**Fig. 5:**
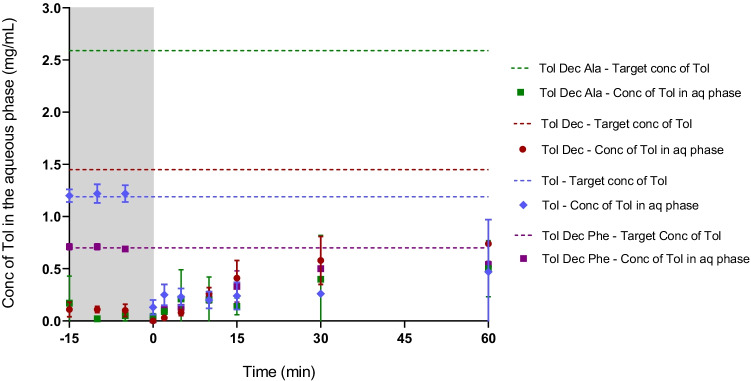
*In vitro* digestion studies of Tol ILs in MCF (Type IIIA) (37°C, mean =  ± SD, n = 3); tolfenamic acid (blue diamond), Tol Dec (red circle), Tol Dec Ala (green square), and Tol Dec Phe (purple square). The dashed line represents the target (100% solubilised) concentration of tolfenamic acid for each formulation. Differences in the solubility of tolfenamic acid and the different Tol ILs dictated that the drug loading of Tol ILs in MCF was different in each case and therefore the target concentrations were different in each case. Concentrations are shown during dispersion (-15 – 0 min; grey section), and digestion (0 – 60 min) of the lipolysis study

When the data is viewed as the concentration of tolfenamic acid in the aqueous phase (Fig. 5), rather than the proportional distribution (Fig. 4), a composite view is obtained that considers both the proportional distribution in Fig. 4 and the drug loading in Table I. These data show that the aqueous phase concentration is highest on formulation dispersion for tolfenamic acid and the Tol Dec Phe IL, in spite of the low drug loading levels. This reflects the lack of phase separation of the dense oil phase seen for Tol Dec and Tol Dec Ala. On digestion, however, the aqueous phase concentrations for the tolfenamic acid and Tol Dec Phe formulations drop rapidly such that immediately after initiation of digestion, the aqueous phase concentration of tolfenamic acid is low and similar across all formulations. As described above, over the digestion period a proportion of the dose that is initially abstracted into the dense oil phase is released to the aqueous phase. As shown in Fig. 5, the highest aqueous phase concentrations are ultimately obtained for the non-digestible Tol Dec IL in the formulation which combines reasonable solubility in the formulation with good transfer to the aqueous phase. In contrast, Tol Dec Ala which showed the highest lipid solubility, also precipitated more readily during formulation digestion and as such the aqueous phase concentrations at the end of the digestion phase were lower than formulations containing Tol Dec. For the ILs where drug loading was enhanced, and therefore where ultimate benefit was initially expected to be highest (e.g. Tol Dec Ala), aqueous phase drug concentrations were limited by the phase separation of an IL-rich oil phase. Since this occurred even during formulation dispersion (at least for the formulations where the IL were loaded at the highest levels), subsequent studies focused on examining whether the formulation characteristics could be changed to improve dispersion of the drug rich formulations.

### Lipolysis studies of Tol ILs in Type IIIB formulations

To improve the dispersion of LBFs containing Tol ILs at high drug loading capacity, i.e. > 10% mass of IL/mass of LBF, the composition of MCF-1 was changed. The proportion of the surfactant, Kolliphor® EL, was increased by twofold and lipid content decreased by 0.5-fold. This results in a change to a more typical Type IIIB formulation, referred to here as MCF-2 (see Table I). The equilibrium solubility of both tolfenamic acid and Tol Dec Ala was increased in MCF-2, likely as a result of the higher surfactant concentration, however the Dec Ala IL retained its solubility advantage in MCF-2, as it had in MCF-1.

The increased quantity of surfactant in MCF-2 was expected to facilitate improved dispersion, and thereby solubilisation of Tol IL. To examine this, Tol ILs were loaded into MCF-2 at molar equivalent concentrations to that employed in the lipolysis studies with MCF-1 and digested as before. The new formulation containing higher concentrations of Kolliphor® EL led to much improved solubilisation of tolfenamic acid. In contrast to the previous lipolysis studies of the Tol ILs in MCF-1, the lipolysis samples of tolfenamic acid, Tol Dec, Tol Dec Phe and Tol Dec Ala did not form a dense oil phase, presumably reflecting the ability of the higher quantities of Kolliphor® EL to better solubilise and disperse the formulation and digestion products.

**Fig. 6:**
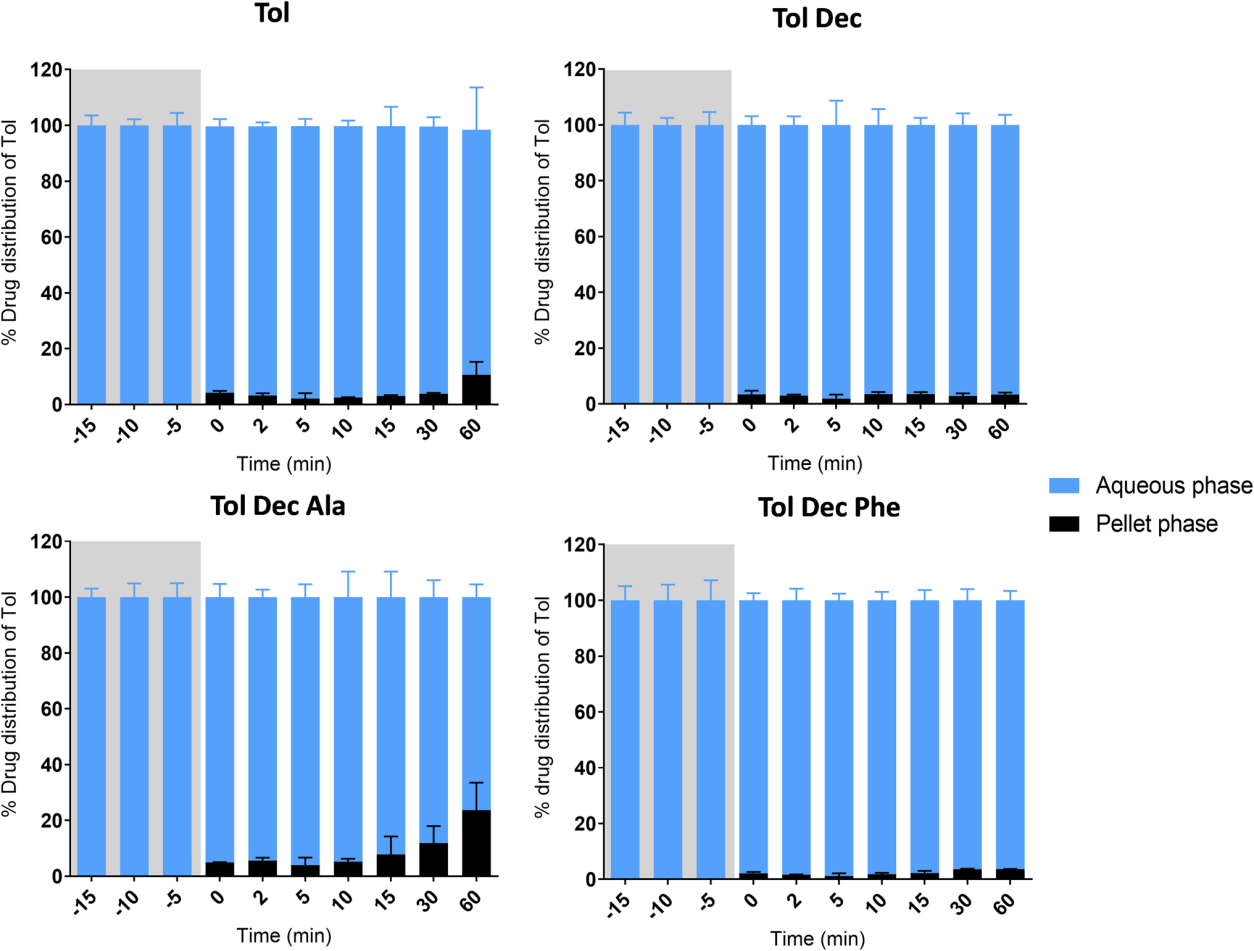
Drug distribution of tolfenamic acid and Tol ILs in MCF-2 (Type IIIB) during *in vitro* digestion studies (37°C, mean ± SD, n = 3). % drug distribution of tolfenamic acid was determined via the concentration of tolfenamic acid in respective phases during dispersion (-15 – 0 min; grey section), and digestion (0 – 60 min)

**Fig. 7:**
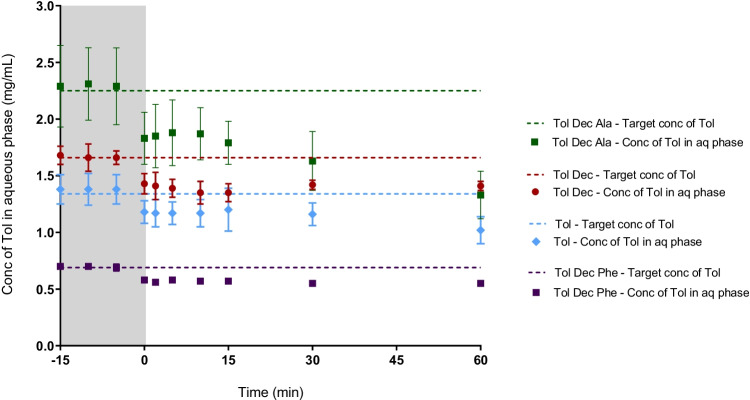
*In vitro* digestion studies of Tol IL in MCF-2 (Type IIIB) formulation. Increasing the proportion of surfactant (Kolliphor® EL) in the formulation, improved formulation dispersion (-15 – 0 min; grey section), and drug solubilisation during digestion (0 – 60 min). Tol Dec and Tol Dec Ala, were loaded into the Type IIIB formulation, at mol equivalent concentrations to that employed in the lipolysis studies with the Type IIIA formulation (MCF-1). (mean ± SD; n = 3)

As shown in Fig. 6, lipolysis samples of tolfenamic acid, Tol Dec_,_ Tol Dec Phe and Tol Dec Ala loaded formulations revealed a translucent aqueous phase and a (limited) pellet phase on centrifugation. The highest aqueous phase concentrations were therefore obtained with Tol Dec Ala due to its higher solubility in the formulation (and therefore higher formulation loading). Comparison of the aqueous phase concentration attained (Fig. 7) relative to the data for the original formulation in Fig. 5 shows that the new MCF-2 formulations of tolfenamic acid, Tol Dec_,_ Tol Dec Phe and Tol Dec Ala resulted in aqueous phase concentrations similar to their targeted maximal possible concentrations of Tol in the aqueous phase on dispersion. On digestion these concentrations dropped slightly, especially for Tol Dec and Tol Dec Ala, but the attained aqueous phase concentrations were significantly higher than after digestion of MCF-1. Whether this reflects concurrent digestion of the LAA counter ion remains unknown. Nonetheless, the data show that when reformulated to provide for adequate dispersion, the MCT-2 Tol Dec Ala formulation provides for the highest attainable drug concentrations after *in vitro* formulation digestion (Fig. 7) This reflects both higher drug loading in the formulation of Tol Dec Ala, due to higher solubility of the ionic liquid, and effective solubilisation post dispersion by virtue of using MCF-2.

### Oral exposure of Tolfenamic acid after administration of Tolfenamic acid and Tol Dec Ala IL in MCF-2

**Fig. 8:**
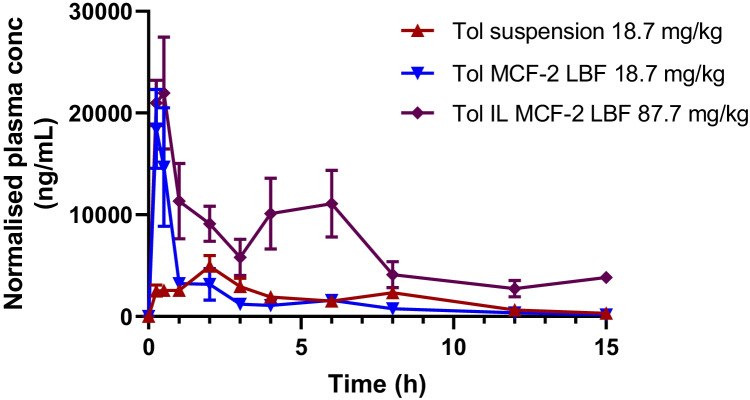
Plasma level time profiles for tolfenamic acid after oral gavage of a suspension and LBF of tolfenamic acid loaded at a dose of ~ 5.6 mg per rat in comparison to the same LBF loaded with ~ 26 mg of the more lipid soluble Tol Dec Ala IL (mean ± SE, n = 3–6). Data normalised to dose of 18.7 or 87.7 mg/kg within each dosing group to account for variation in animal weights

In light of the *in vitro* data indicating that the Dec Ala IL counterion was both digestible and resulted in higher drug loading capacity in LBF than Tol Dec, and that coupling this with MCF-2 (but not MCF-1) led to higher tolfenamic acid solubilisation during formulation dispersion and digestion for MCF-2 containing Tol Dec Ala compared to Tol Dec (Fig. 7), subsequent studies explored the *in vivo* utility of the MCF-2 – Tol Dec Ala formulation. Tolfenamic acid and Tol Dec Ala were loaded into MCF-2 at concentrations close to their maximum solubility and dosed via oral gavage to conscious rats. As a comparator, a lipid free oral suspension of tolfenamic acid was also administered at the same dose as that given in MCF-2. Plasma samples were taken over time and assayed for tolfenamic acid by HPLC–MS/MS. The plasma level time profiles are shown in Fig. 8 and the summary pharmacokinetic data summarised in Table II.

**Table II:** Summary pharmacokinetic parameters for tolfenamic acid in plasma after oral gavage of suspension and lipid-based formulations (MCF-2) of tolfenamic acid and a lipid-based formulation (MCF-2) of Tol Dec Ala ionic liquid

Compound	Formulation	Dose (mg/kg Tol equivalents)	T_max_ (h)	C_max_ (µg/mL)	AUC _(0-15 h)_ (µg/mL.h)	AUC _(0-15 h)_ (µg/mL.h) normalised*
Tolfenamic acid	Suspension	18.7	1.19 ± 0.88	5.3 ± 1.9	27.2 ± 6.85	27.2 ± 6.85
Tolfenamic acid	LBF (MCF-2)	18.7	0.33 ± 0.14	20.6 ± 6.0	30.5 ± 9.82	30.5 ± 9.82
Tol Dec Ala IL	LBF (MCF-2)	87.7	0.33 ± 0.13	21.7 ± 11	95.5 ± 46.1	38.8 ± 18.7

* Data normalised to a nominal dose of 18.7 mg/kg Tol to provide dose normalised exposure comparison.

The plasma level time data show that administration in a lipid-based formulation leads to a more rapid T_max_ for formulations of Tol than administration as an oral suspension, but that no significant difference is evident in total exposure (AUC). This was expected and is consistent with previous studies employing tolfenamic acid as a model poorly water-soluble anion and likely reflects the fact that whilst the aqueous solubility of the free acid of tolfenamic acid is low, under intestinal conditions at neutral pH, where Tol is expected to be ionised, the solubility is less likely to limit absorption. Reflecting the greater lipid solubility of the Tol Dec Ala IL, LBF containing this IL could be loaded with larger quantities of drug. The larger quantities dosed resulted in a significant increase in oral exposure. In spite of the larger dose, Tol exposure after oral administration of MCF-2 containing Tol Dec Ala increased proportionally and oral bioavailability was maintained as shown by the consistency of the dose normalised AUC data across all formulations. Some evidence of double peaking was apparent after administration of the Tol Dec Ala IL. This may reflect enterohepatic recycling as this has been suggested previously [90, 91], although it was less evident in the other dosing groups. It may also reflect the administration of a higher dose, requiring longer to absorb, possibly coupled with gradual digestion of the LAA counterion.

## Conclusion

Pairing tolfenamic acid, a weakly acidic drug with a cationic LAA produced a lipophilic drug-IL with improved drug loading capacity in LBF compared to the parent drug. This approach is applicable to a range of weakly acidic small molecules and is expected to improve the utility of LBF and reduce pill burden or offer reduced capsule sizes to patients. Cationic lipophilic salt counterions, however, are challenging pharmaceutically since most cationic lipids have toxicity liabilities. In the current studies LAA were therefore employed in order to introduce a digestible bond into the cationic lipid. Consistent with this hypothesis, data obtained under model intestinal conditions showed very rapid breakdown under digestion conditions to produce less toxic fatty alcohols and amino acids. Initial attempts to employ Type IIIA medium chain lipid-based formulations to formulate the LAA-based ILs were unsuccessful when loaded at the high drug levels that were possible using the ILs. However, substitution of the Type IIIA formulations with a similar Type IIIB formulation with higher surfactant levels resulted in improved performance and good solubilisation throughout the experiment. Subsequently oral bioavailability studies in rats demonstrated that the same Type III LBF containing the Tol Dec Ala IL was able to support effective oral exposure even at the higher drug loading level facilitated by IL conversion. The data suggest that cationic LAAs can serve as digestible lipid cations for the formation of highly lipid soluble ILs from weakly acidic drugs, and provides proof-of-concept for a novel series of biocompatible lipid cations suitable for *in vivo* use.

## Supplementary Information

Below is the link to the electronic supplementary material.

Supplementary file1 (DOCX 914 KB)
