# Supplementary material for: Biocompatible Cationic Lipoamino Acids as Counterions for Oral Administration of API-Ionic Liquids
Source: Pharm Res. 2022 Jun 3;39(10):2405–19. doi: 10.1007/s11095-022-03305-y (PMC9556374; doi:10.1007/s11095-022-03305-y)

# **Supplementary Information**

# **Biocompatible cationic lipoamino acids as counterions for oral administration of API-ionic liquids**

Anthony Lai^a,b^, Nathania Leong^b^, Dan Zheng^b^, Leigh Ford^a,c^, Tri-Hung Nguyen^b^, Hywel D. Williams^b,d^, Hassan Benameur^b^, Peter J. Scammells^a^ and Christopher J. H. Porter^b*^

Affiliations

a. Medicinal Chemistry, Monash Institute of Pharmaceutical Sciences, Monash University, 381 Royal Parade, Parkville, Victoria, 3052 Australia

b. Drug Delivery, Disposition and Dynamics, Monash Institute of Pharmaceutical Sciences, Monash University, 381 Royal Parade, Parkville, Victoria, 3052 Australia.

c. Current Address Uniquest, General Purpose South Building, Staff House Rd, The University of Queensland QLD 4072 Australia

d. Current Address CSL Limited, 45 Poplar Road, Parkville, Victoria 3052, Australia.

* Corresponding author Email: [chris.porter@monash.edu](mailto:chris.porter@monash.edu)

## **Chemical Characterisation**

### **Tolfenamate decyl amine (Tol Dec)**

^1^H NMR (DMSO-d6) δ 7.89 (dd, *J* = 7.7, 1.6 Hz, 2H), 7.30 (d, *J* = 8.0 Hz, 1H), 7.12 (dt, *J* = 16.0, 8.1 Hz, 2H), 7.02 (d, *J* = 8.4 Hz, 2H), 6.97 (d, *J* = 7.7 Hz, 2H), 6.66 (t, *J* = 7.8 Hz, 1H), 2.81 – 2.71 (m, 2H), 2.28 (s, 3H), 1.51 (d, *J* = 7.4 Hz, 2H), 1.23 (s, 14H), 0.85 (t, *J* = 6.8 Hz, 3H). ^13^C NMR (101 MHz, DMSO-d6) δ 170.10 (C=O, cation), 131.86 (CH, cation), 129.74 (CH, cation), 127.07 (CH, cation), 120.98 (CH, cation), 117.19 (CH, cation), 115.85 (CH, cation), 113.89 (CH, cation), 31.28 (CH_2_, anion), 28.86 (CH_2_, anion), 28.67 (CH_2_, anion), 25.92 (CH_2_, anion), 22.08 (CH_2_, anion), 14.58 (CH_3_, cation), 13.96 (CH_3_, anion). HRMS (m/z) ^+^ve mode: calculated for C_10_H_24_N^+^; 158.1902, found; 158.1903 (0.95 ppm). HRMS (m/z) ^–^ve mode: calculated for C_14_H_11_NO_2_^-^; 260.0491, found; 260.0484 (-2.82 ppm). Yield = 99%. Purity = 99%. M.P = 137 – 143°C.

### **Tolfenamate decyl alanine ester (Tol Dec Ala)**

^1^H NMR (DMSO-d6) δ 7.91 (d, *J* = 7.3 Hz, 1H), 7.38 – 7.02 (m, 4H), 6.94 (d, *J* = 8.2 Hz, 1H), 6.72 (t, *J* = 7.4 Hz, 1H), 4.07 (dq, *J* = 10.8, 6.5 Hz, 2H), 3.85 (dd, *J* = 14.0, 7.0 Hz, 1H), 2.27 (s, 3H), 1.66 – 1.47 (m, 2H), 1.45 – 1.09 (m, 17H), 0.85 (t, *J* = 6.5 Hz, 3H). ^13^C NMR (101 MHz, DMSO-d6) δ 170.10 (C=O, cation), 146.60 (C=O, anion), 134.43 (CH, cation), 128.14 (CH, cation), 127.77 (CH, cation), 123.29 (CH, cation), 119.23 (CH, cation), 117.74 (CH, cation), 114.12 (CH, cation), 65.38 (CH_2_, anion), 48.87 (CH, anion), 31.76 (CH_2_, anion), 29.39 (CH_2_, anion), 29.15 (CH_2_, anion), 29.08 (CH_2_, anion), 28.48 (CH_2_, anion), 25.69 (CH_2_, anion), 22.57 (CH_2_, anion), 18.05 (CH­_3_, anion), 15.10 (CH_3_, cation), 14.43 (CH_3_, anion). HRMS (m/z) ^+^ve mode: calculated for C_13_H_28_NO_2_^+^; 230.2121, found; 230.2115 (-2.74 ppm). HRMS (m/z) ^–^ve mode: calculated for C_14_H_11_NO_2_^-^; 260.0478, found; 260.0484 (2.32 ppm). Yield = 70%. Purity = 99%. M.P = 90 – 178°C.

### **Tolfenamate decyl phenylalanine ester (Tol Dec Phe)**

^1^H NMR (DMSO-d6) δ 7.97 – 7.81 (m, 1H), 7.40 – 7.06 (m, 9H), 6.89 (d, *J* = 8.4 Hz, 1H), 6.74 (t, *J* = 7.2 Hz, 1H), 3.96 (td, *J* = 6.5, 3.5 Hz, 2H), 3.75 (t, *J* = 6.8 Hz, 1H), 2.88 (d, *J* = 6.9 Hz, 2H), 2.26 (s, 3H), 1.53 – 1.34 (m, 2H), 1.25 (d, *J* = 15.3 Hz, 14H), 0.85 (t, *J* = 6.8 Hz, 3H). ^13^C NMR (101 MHz, DMSO-d6) δ 170.10 (C=O, cation), 147.52 (C=O, anion), 132.72 (CH, cation), 130.12 (CH, anion), 129.13 (CH, anion), 128.29 (CH, cation), 127.48 (CH, cation), 124.51 (CH, cation), 120.96 (CH, cation), 118.16 (CH, cation), 114.50 (CH, cation), 65.38 (CH_2_, anion), 61.61 (CH, anion), 55.66 (CH, anion), 32.18 (CH_2_, anion), 29.81 (CH_2_, anion), 29.77 (CH_2_, anion), 29.58 (CH_2_, anion), 29.49 (CH_2_, anion), 28.83 (CH_2_, anion), 26.10 (CH_2_, anion), 22.99 (CH_2_, anion), 15.55 (CH_3_, cation), 14.84 (CH_3_, anion). HRMS (m/z) ^+^ve mode: calculated for C_19_H_32_NO_2_^+^; 306.2423, found; 306.2428 (1.51 ppm). HRMS (m/z) ^–^ve mode: calculated for C_14_H_11_NO_2_^-^; 260.0487, found; 260.0484 (-1.42 ppm). Yield = 80%. Purity = 99%. M.P = 88 – 174°C.

## **NMR**


### **Tol Dec**


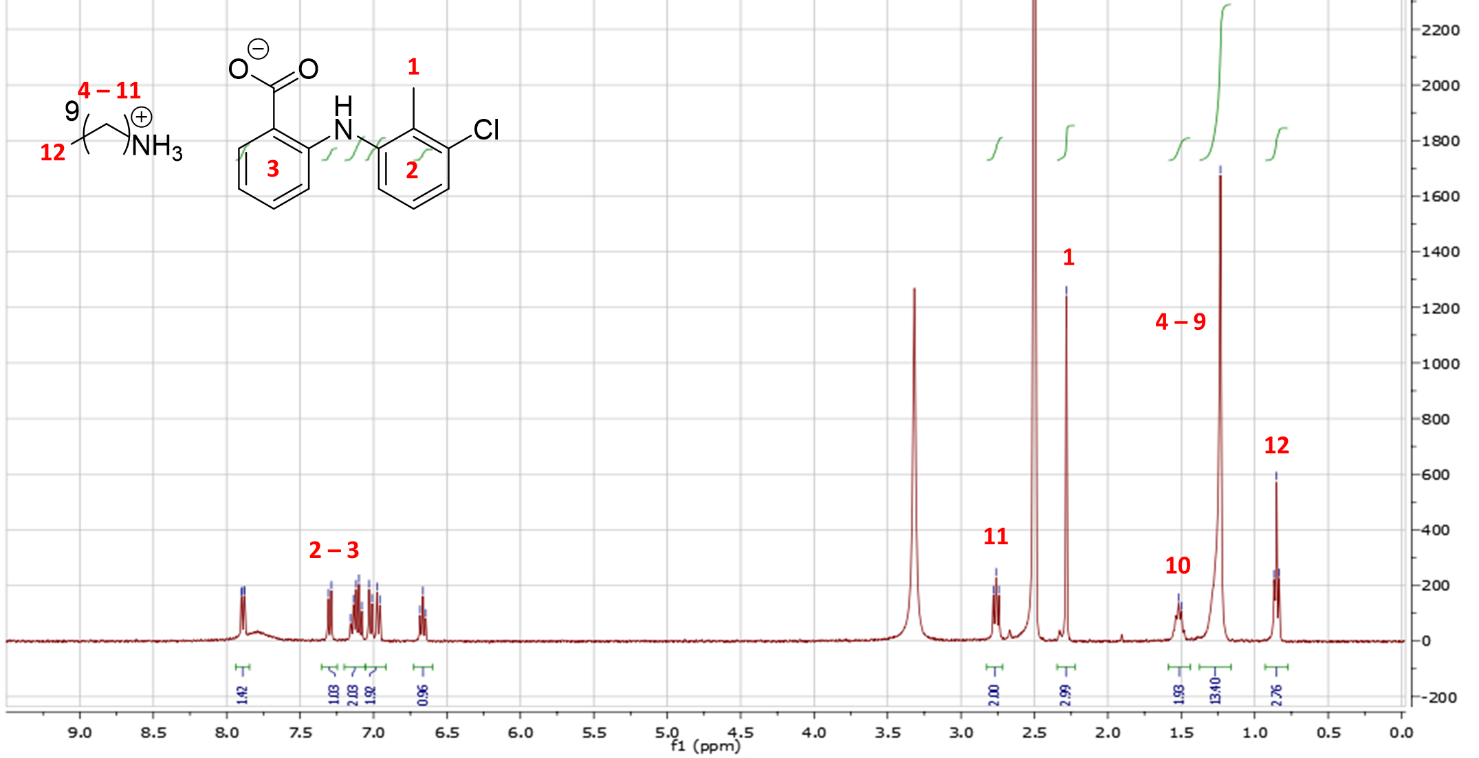


###

### **Tol Dec Ala**


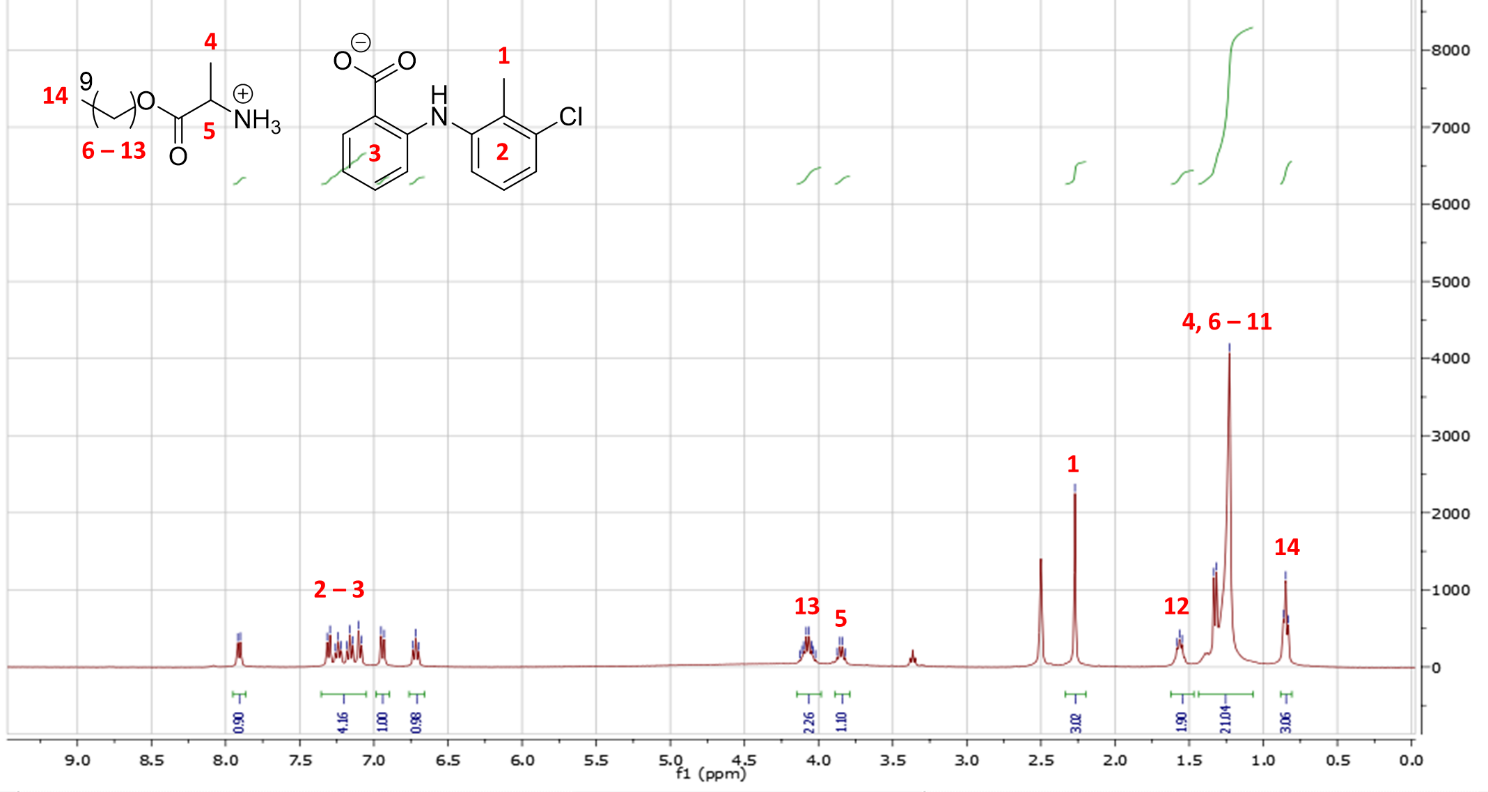


### **Tol Dec Phe**


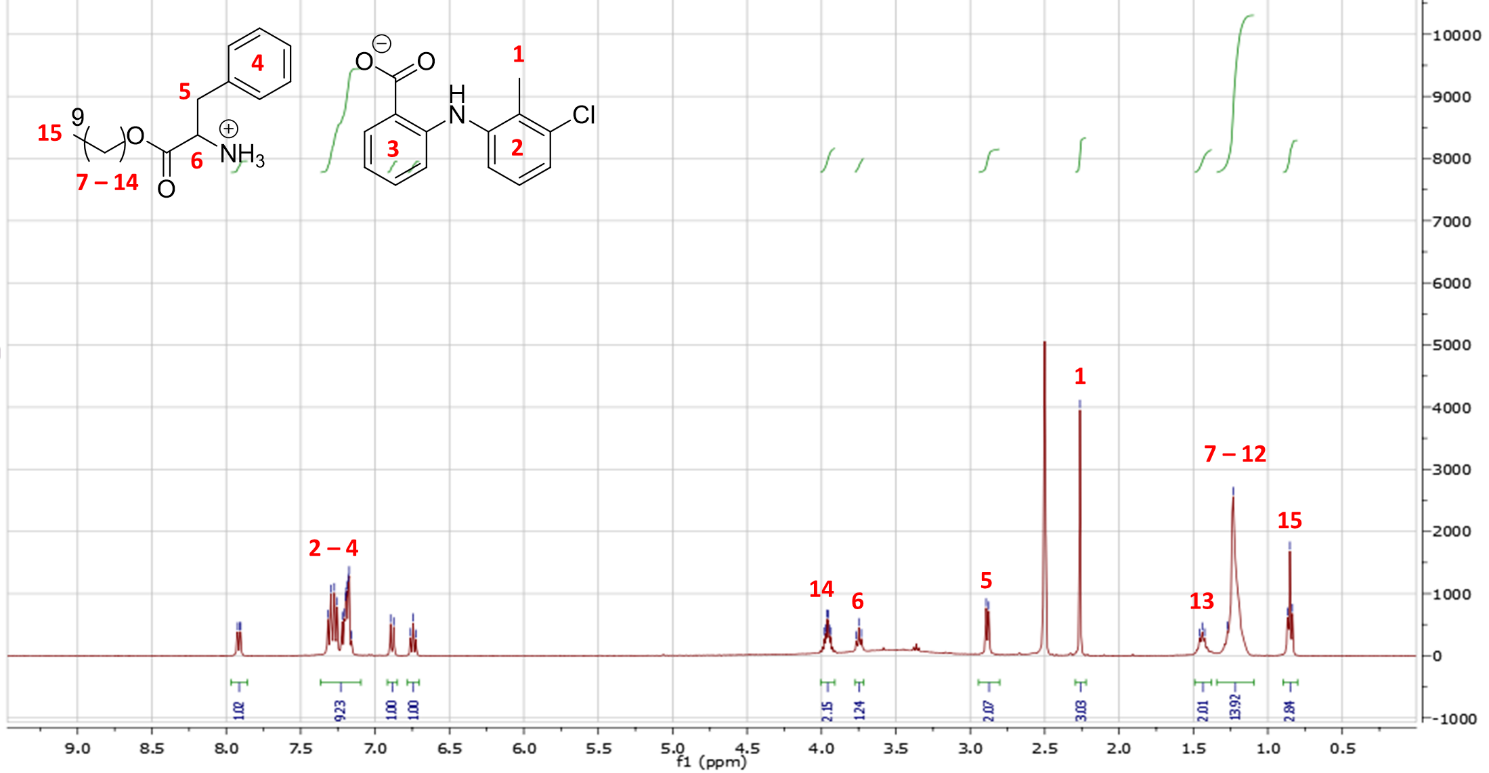


**DSC Characterisation**

Samples (2-10 mg) were directly weighed into aluminium pans and either hermetically sealed (Tol Dec) or used with perforated (pin hole) lids (Tol Dec Ala, Tol Dec Phe). Differential scanning calorimetry (DSC) analysis was performed using a PerkinElmer DSC 8500 (Waltham, MA) with Intracooler III integrated cooler. During the analysis, sample chambers were continually flushed under nitrogen (20 mL/min) and an empty aluminium pan was used to establish the baseline as a reference control. Samples were analysed in one heating cycle (at either 10 ^o^C/min (Tol Dec) from 50 ^o^C to 170 ^o^C or 20 ^o^ C/min (Tol Dec Ala, Tol Dec Phe) from -50 ^o^C to 150^o^C.

**
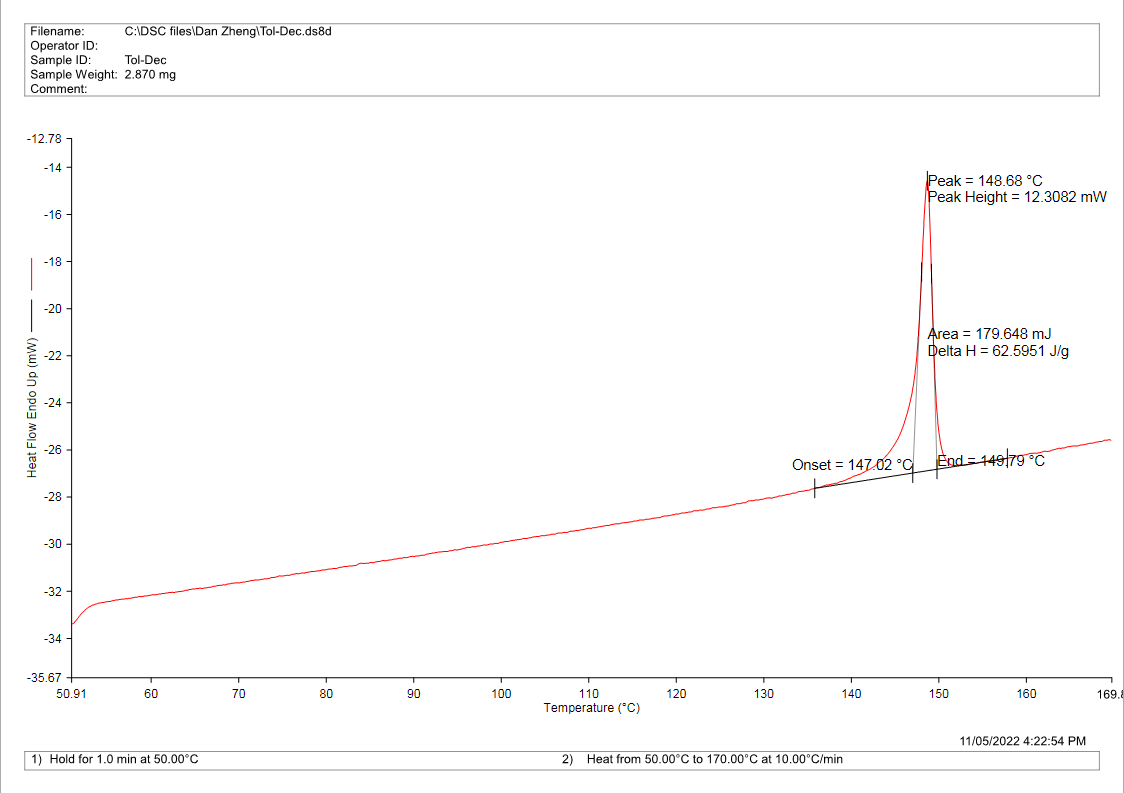
DSC for Tol Dec**

**
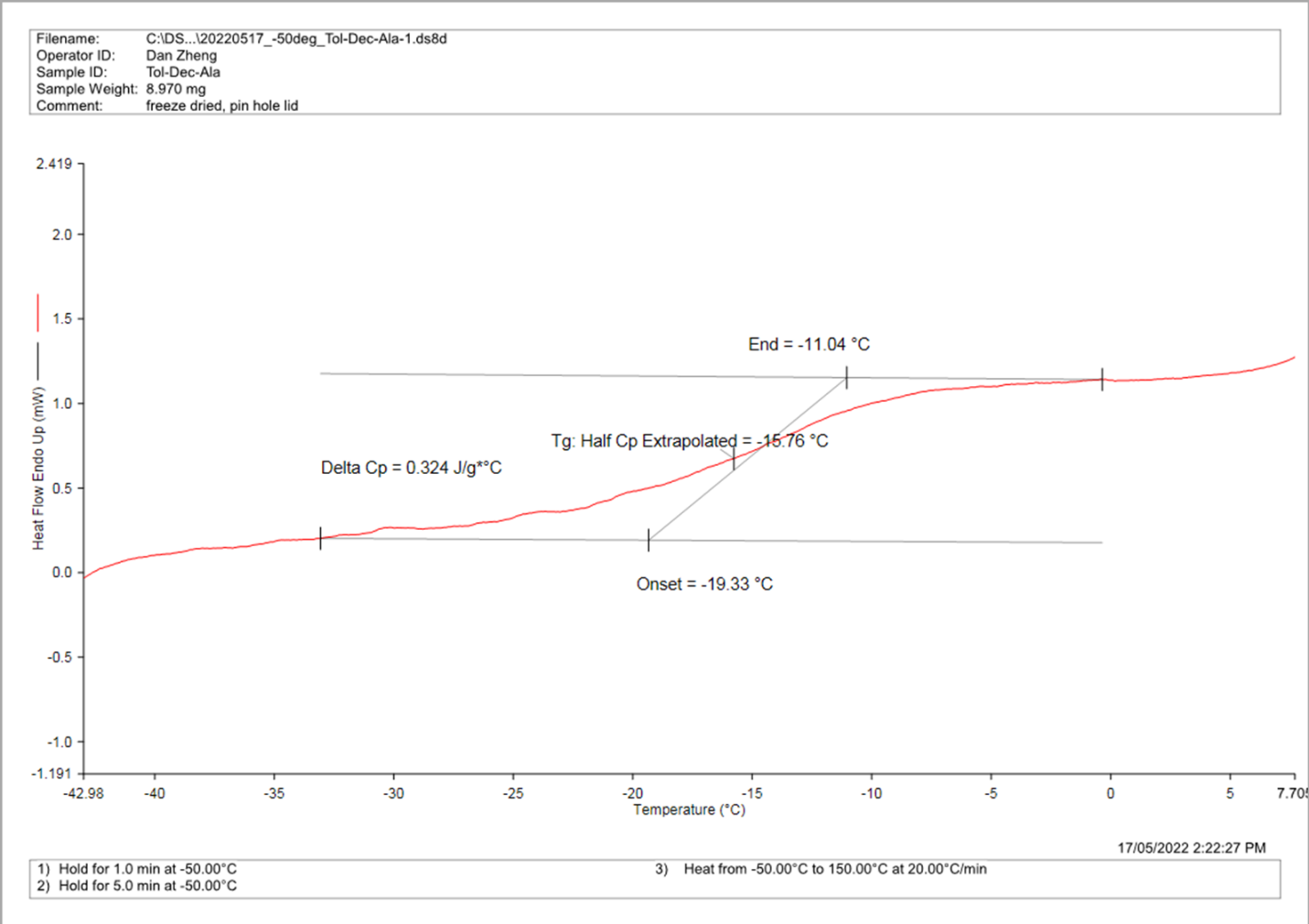
DSC for Tol Dec Ala**

**DSC for Tol Dec Phe**


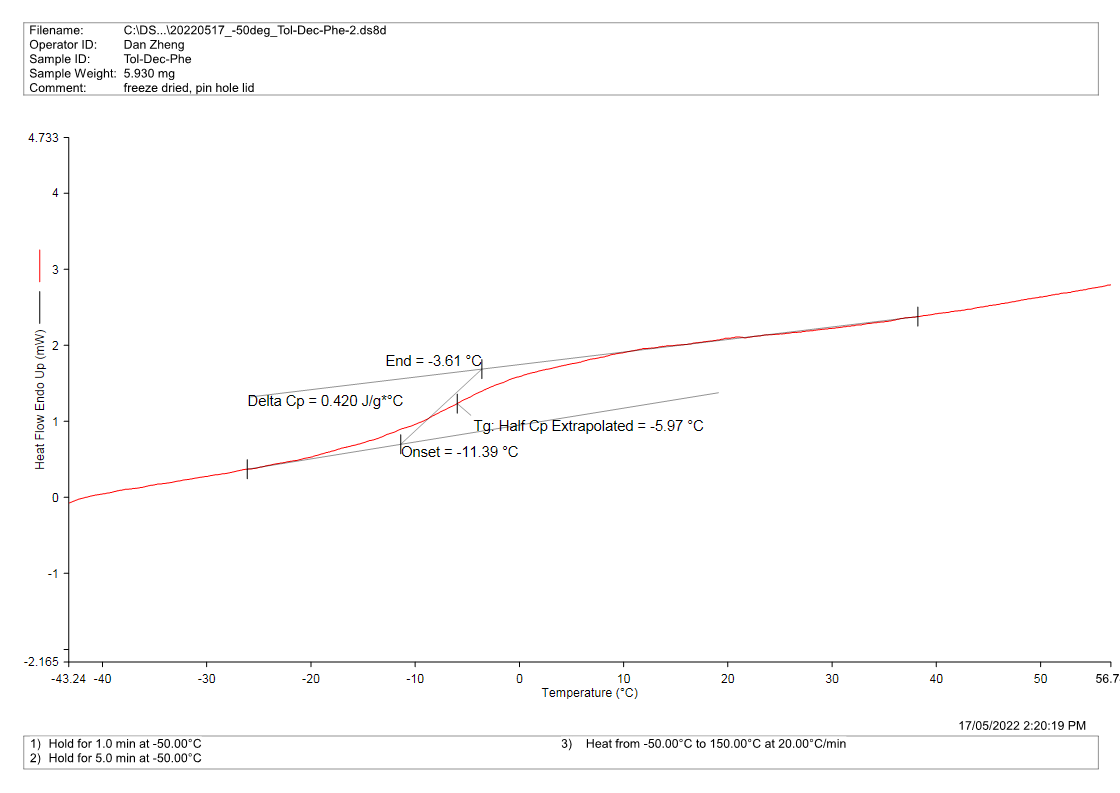

Supplement: Supplementary file 1 — Supplementary file1 (DOCX 914 KB) [file 11095_2022_3305_MOESM1_ESM.docx]
